# Clinical Analysis of Congenital Deficient Tracheal Cartilage Rings: Six Case Reports and a Literature Review

**DOI:** 10.3389/fped.2020.548089

**Published:** 2020-10-20

**Authors:** Jing Ma, Chen Meng, Zhiyu Feng, Xiaorong Han, Shuaishuai Liu, Na Liu, Weiwei Zhu

**Affiliations:** ^1^Department of Pediatrics, Jinan Central Hospital, Cheeloo College of Medicine, Shandong University, Jinan, China; ^2^Department of Respiratory Intervention, Qilu Children's Hospital of Shandong University, Jinan, China; ^3^Department of Cardiac Surgery, Qilu Children's Hospital of Shandong University, Jinan, China

**Keywords:** congenital tracheal stenosis, tracheal reconstruction, tracheal rings deficiency, bronchoscopy, pediatric stridor, airway obstruction, airway stent

## Abstract

Congenital deficiency of tracheal rings is a rare tracheal malformation that can cause central airway obstruction. Herein we reported the clinical data of six patients with symptomatic congenital deficient tracheal rings. There were five cases, with isolated short-segment absent cartilage ring located on the distal trachea (three cases), cervical trachea (one case), and distal trachea combined with bilateral bronchi (one case). Among them, four (4/5) received surgical tracheal resection, three fully recovered, and one died of severe infection. Besides that, one patient, who could not be weaned off the mechanical ventilation, died after rejecting surgery. One case had episodes of recurrent dyspnea and extubation failure due to long-segment tracheomalacia after repair of esophageal atresia and tracheoesophageal fistula. For this patient, deficient cartilage rings were suspected and confirmed at the age of 26 months. Moreover, the clinical characteristics of 12 cases with congenital deficient tracheal cartilage rings reported in previous literature were reviewed. The different characteristics between short- and long-segment deficient cartilage rings were discussed.

## Introduction

Congenital tracheal anomalies can be caused by abnormal cartilage rings or pars membranacea. In broad terms, congenital anomalies of tracheal rings include tracheal agenesia, deficient/absent cartilage rings, and tracheomalacia (1). Congenital tracheomalacia is defined as increased collapsibility of the trachea. The majority of children with congenital tracheomalacia have mild to moderate symptoms and do not need surgical intervention considering that the soft trachea becomes more rigid with growth (2). On the other hand, deficient or even absent cartilage rings are an extremely rare anomaly that can be easily misdiagnosed with stenosis or malacia. In 1991, Santoli (3) reported a case of tracheomalacia in a 6-month-old baby with severe symptoms, yet after performing surgery at the age of 5 years, absence of cartilage ring was confirmed by histological examination of the resected trachea.

Over recent years, a small number of cases with congenital deficient tracheal cartilage rings with different characteristics and complications have been reported. Herein we reported the clinical data of six children with congenital deficient tracheal rings. In addition, the clinical characteristics of 12 cases with congenital deficient tracheal cartilage rings reported in previous literature were reviewed.

### Case Presentation

Six children (five males and one female) with an average age ranging from 1 to 12 months were diagnosed with a congenital deficiency of tracheal rings between July 2017 and May 2020. Segmental cartilage rings, with a length of 1–1.5 cm, were absent in five babies. One case with VACTERL syndrome manifested esophageal-like lumen with no cartilage rings in the middle trachea and was misdiagnosed as severe tracheomalacia. Clinical presentations, imaging manifestations, bronchoscopy, interventional treatment, and follow-up results are summarized in Table 1.

**Table 1:** Clinical data of six children with deficient tracheal rings.

**No**.	**Gender**	**Episode/diagnosis age**	**Clinical manifestation**	**Endoscopic performance**	**Associated abnormalities**	**Treatment**	**Postoperative endoscopic examination**	**Interventional therapy**	**Follow-up time**	**Follow-up result**
1	M	4 months/1 year	Repeated wheezing	1-cm segmental concentric stenosis at the distal trachea	No	Tracheal resection + tracheoplasty at the age of 1 year	Granulation and anastomotic restenosis 25 days after surgery	Laser therapy + balloon dilatation	24 months	Survive, asymptomatic
2	F	Postnatal/8 months	Repeated stridor	1.5-cm segmental concentric stenosis at the distal trachea	Acleistocardia	Tracheal resection + end-to-end anastomosis at the age of 9 months	Surgical sutures in the lumen	Laser therapy	14 months	Survive, asymptomatic
3	M	Postnatal/2 months	Repeated wheezing	1-cm segmental concentric stenosis at the cervical trachea	Acleistocardia	Tracheal resection + slide tracheoplasty at the age of 7 months	Bilateral vocal cord paralysis, anastomotic scar stenosis, a small amount of granulation hyperplasia	Laser therapy	16 months	Survive, mild stridor
4	M	Postnatal/3 months	Dyspnea under intubation and mechanical ventilation	Stenosis in distal trachea and bilateral bronchi opening	No	Tracheal resection + carinaplasty + end-to-end anastomosis at the age of 3 months	Anastomotic infection and rupture	Reoperation	21 days	Death
5	M	1/2 months	Dyspnea	1-cm segmental stenosis at the distal trachea	Acleistocardia	Mechanical ventilation, refuse surgery	–	–	20 days	Death
6	M	Postnatal/2 months	Dyspnea and failed extubation	The severe collapse of the middle to distal trachea	Esophageal atresia, tracheoesophageal fistula, solitary kidney, acleistocardia	7–8 × 25-mm Dumon stent placement	Stent migration after 5 months	Stent removal and replacement with 9–10 × 37-mm stent	43 months	Survive with stent

*M, male; F, female*.

### Case 1

The patient was a 1-year-old boy who experienced two episodes of wheezing. He was admitted due to repeated cough and wheezing. The symptoms were not relieved after antibiotic treatment and inhalation of a bronchodilator. He was transferred to continuous positive airway pressure (CPAP) therapy. Chest CT demonstrated short-segmental stenosis in the distal trachea, while no abnormalities were found in the major cardiopulmonary vessels. On the 8th day after admission, bronchoscopy revealed segmental dynamic concentric stenosis of about 0.5 cm proximal to the carina. Subsequently, a 1-cm-long section without cartilage ring was resected, and tracheoplasty was performed under cardiopulmonary bypass. Decannulation was performed 1 day later. On the 25th day after the operation, bronchoscopy showed granulation hyperplasia and anastomotic restenosis, which was relieved by laser ablation and balloon dilation. A review of the bronchoscopy at 16 months after the operation showed that the lumen was slightly narrow and permitted the passage of an endoscope with an outside diameter of 4.0 mm. Presently, the boy is 3 years old and asymptomatic, with normal growth and development.

### Case 2

The second patient was a 1-year-old girl who had stridor after birth. At the age of 8 months, she had episodes of recurrent wheezing and respiratory infections. Bronchoscopy showed segmental stenosis 0.5 cm proximal to the carina, which was without cartilage rings. Chest CT airway reconstruction suggested distal tracheal stenosis, while it did not confirm anomalies in major vessels. Cardiac ultrasound showed acleistocardia. Surgery was performed 1 month later. Segmental soft stenosis was found at 0.5 cm proximal to the carina, with a length of about 1.5 cm; the cartilage ring of stenosis was absent. The segment was resected, followed by end-to-end anastomosis under cardiopulmonary bypass. Bronchoscopy showed a clear lumen, with a diameter >4.0 mm, 2 months after the surgery. At the opening of the right main bronchus, the surgical suture protruded into the lumen and was removed by forceps after laser ablation. At the end of follow-up, the child was asymptomatic 14 months after the operation.

### Case 3

**Figure 1:**
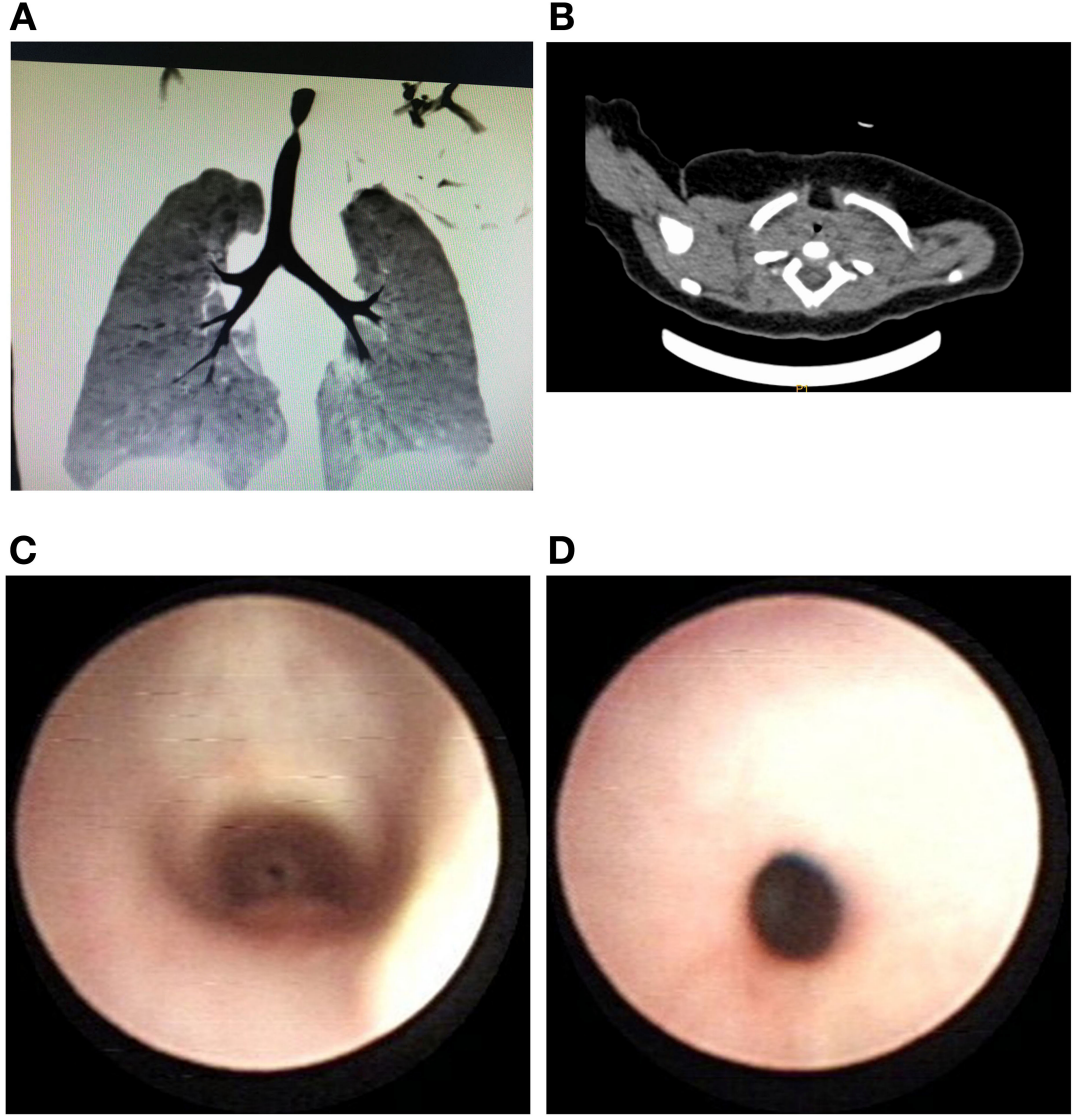
**(A)** Airway reconstruction shows the localized stenosis of the lumen in the cervical trachea. **(B)** The irregular narrow lumen of the cervical trachea shown by a CT scan. **(C)** Cervical stenosis was observed through the subglottic region during flexible bronchoscopy. **(D)** Bronchoscopy showing segmental concentric stenosis.

The boy was admitted to a hospital because of persistent stridor that occurred for the first time at the age of 2 months. Neck and chest CT showed segmental stenosis at the cervical trachea (Figures 1A,B). Bronchoscopy revealed concentric stenosis with absent cartilage rings at the cervical trachea, while the middle trachea and the distal trachea were normal (Figures 1C,D). Tracheal reconstruction surgery was suggested, yet his guardians refused the operation. At the age of 7 months, he was admitted to our hospital due to aggravated wheezing and cyanosis after crying. Tracheal surgery was performed under cardiopulmonary bypass. During the operation, a 1-cm-long stenosis trachea without cartilage rings was resected. Slide tracheoplasty was subsequently performed. Two months after the operation, the patient returned due to progressive cough and wheezing. Bronchoscopy showed bilateral vocal cords paralysis as well as luminal scar contracture and granulation tissue at the anastomotic lumen. His symptoms were alleviated after laser ablation. Eight months after the operation, bronchoscopy showed irregular morphology and mild stenosis, permitting the passage of a 4-mm bronchoscope. At the end of follow-up, the child was 2 years old and asymptomatic at rest but had slight stridor after a respiratory infection, which could be relieved by inhaling budesonide.

### Case 4

**Figure 2:**
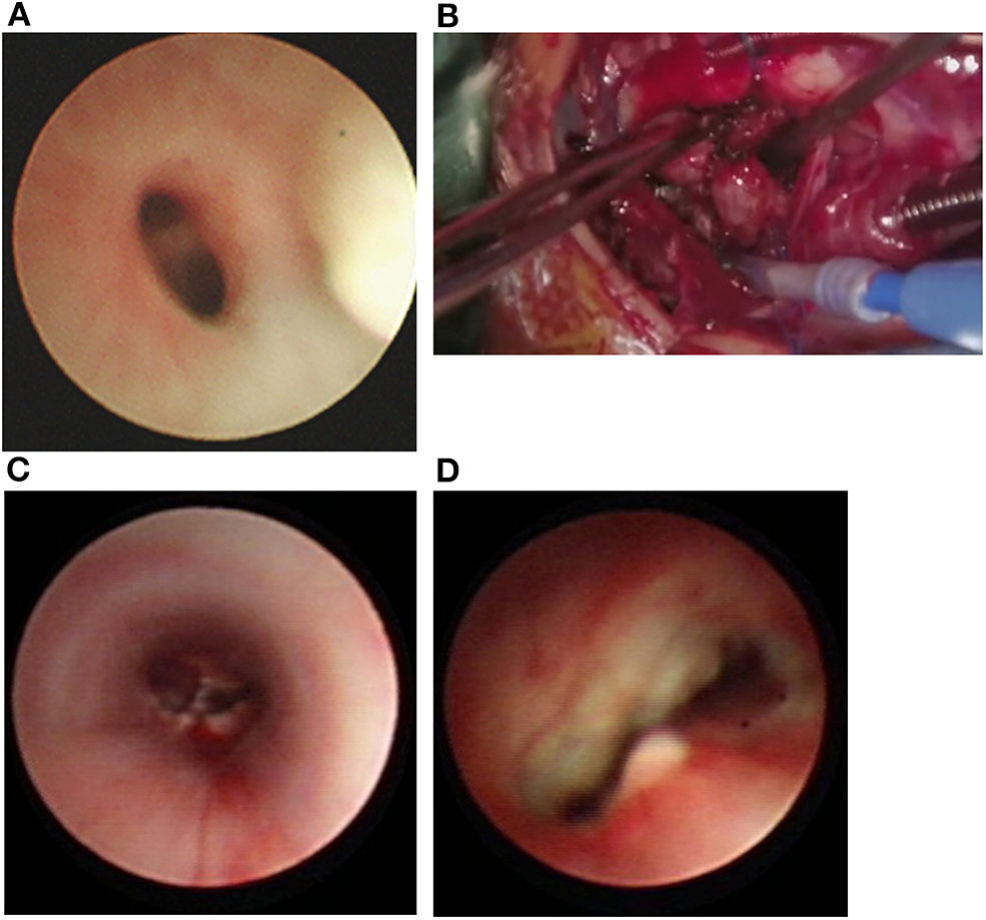
**(A)** Bronchoscopy showing severe stenosis at the distal tracheal and juxa-tacarinal location. **(B)** During operation, soft stenosis without cartilage rings located in the distal trachea and bilateral main bronchi opening. **(C)** Bronchoscopy demonstrated that the carina and bilateral main bronchus were unobstructed after the operation. **(D)** Anastomotic fracture, 9 days after the operation.

The child was a younger twin (34 + 4 weeks) with a birth weight of 1.7 kg. Due to low Apgar scores and respiratory failure, he was intubated for 20 days. At the age of 1 month, he was hospitalized for paroxysmal wheezing and cyanosis. Bronchoscopy at the local hospital revealed obvious stenosis of the distal trachea. His symptoms were progressively aggravated, and he was transferred to our hospital with intubated ventilation at the age of 3 months. Chest CT showed pneumonia and stenosis of the distal trachea and the bronchi at the level of the carina. Hypoxemia could not be corrected under mechanical ventilation. Emergency bronchoscopy showed severe stenosis of the distal trachea at the juxtacarinal location (Figure 2A). He then underwent emergency surgery under cardiopulmonary bypass, which revealed that the distal tracheal and bilateral main bronchi openings were soft and tissue-like, with an extremely narrow diameter of about 1.5 mm (Figure 2B). Tracheal resection and carinaplasty were performed, and no cartilage ring was seen in the excised tissue. After the operation, bronchoscopy showed that the carina and the bilateral bronchi were clear, permitting the passage of a 4-mm bronchoscope (Figure 2C). Alveolar lavage fluid and sputum culture suggested *Acinetobacter baumannii* infection. Bronchoscopy revealed anastomotic fracture 9 days after the operation (Figure 2D). The operation performed on the next day showed that the anterior wall of the reconstructed carina was cracked. The bovine pericardial patch was sutured to repair the cracked area. Cardiotonic, diuretic, and anti-infection drugs (amikacin, cefoperazone-sulbactam, tigecycline, and voriconazole) were given. The child's temperature was unstable, and extubation was not successful. Unfortunately, the patient died 21 days after the surgery.

### Case 5

A 2-month-old boy was admitted due to aggravated wheezing and tachypnea. As progressive tachypnea was not relieved after CPAP for 4 days, he underwent endotracheal intubation at a local hospital. A chest X-ray showed pneumonia. After 16 days of anti-infection and anti-inflammatory therapy, he was weaned off mechanical ventilation and was intubated on the next day again due to dyspnea. Tracheal deformity was suspected. Flexible bronchoscopy demonstrated a 1-cm, soft concentric stenosis at the distal trachea proximal to the carina. He died of respiratory failure after rejecting surgery.

### Case 6

**Figure 3:**
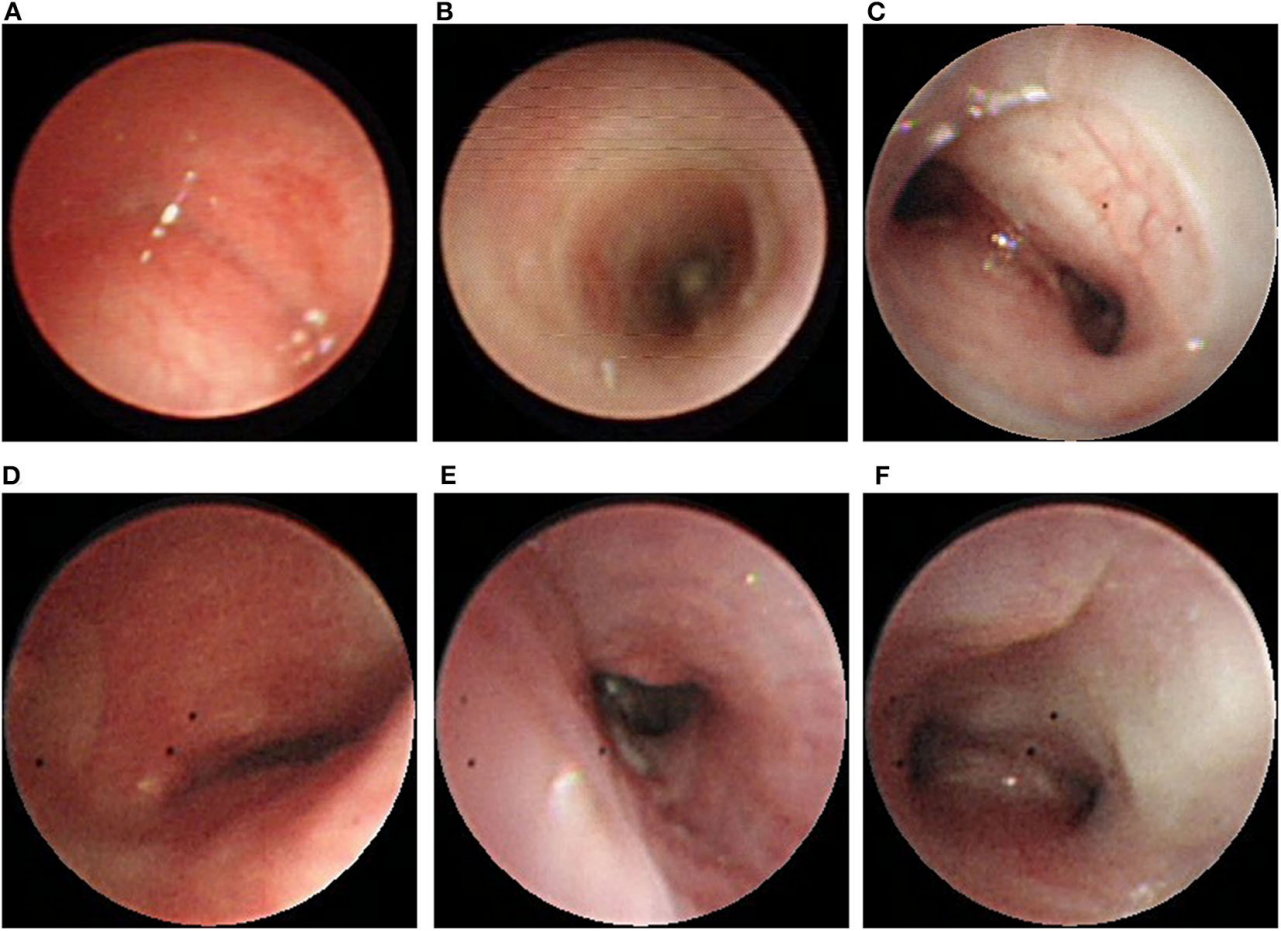
**(A)** Bronchoscopy showing complete collapse at the middle to distal trachea. **(B)** Unblocked trachea after Dumon stent was placed. **(C)** Malacic lumen at the distal trachea and carina. **(D)** Complete collapse without cartilage rings at the middle trachea was observed after stent removal. **(E)** The rigidity of the distal trachea had increased. **(F)** Unobstructed carina after stent removal.

A 35-week premature boy, weighing 3,140 g, was intubated immediately after birth due to severe dyspnea. Esophageal atresia (EA) with tracheoesophageal fistula (TEF), acleistocardia, and solitary kidney were diagnosed. Consequently, VACTERL syndrome was considered. On the 4th day after birth, he underwent end-to-end anastomosis of the esophagus and repair of TEF under the thoracoscope. He suffered extubation failure after surgery, and bronchoscopy showed severe tracheomalacia. The patient was then transferred to our hospital for further treatment at the age of 2 months. Flexible bronchoscopy showed severe malacia of the middle trachea to the distal trachea, and the membranous trachea was widened (Figure 3A). A 7–8 × 25-mm Dumon stent (Novatech S.A.) was placed through the rigid bronchoscope (Figures 3B,C). The patient was decannulated on the next day and received CPAP for 4 days. After 20 days of anti-infection and nutritional support, he was discharged with steady breathing. At the age of 8 months, he was hospitalized for tachypnea and wheezing after crying. Bronchoscopy showed that the stent did not adhere well and migrated; thus, the stent was removed. After the procedure, inspiratory dyspnea occurred again, and the symptoms were not relieved after CPAP. On the next day, 9–10 × 37-mm silicone stents (Novatech S.A.) were implanted through a rigid bronchoscope, and the dyspnea was relieved, yet after the stent was removed, at the age of 26 months, the patient developed dyspnea. A careful reevaluation by flexible bronchoscopy indicated a full collapse of the 1.5-cm esophageal-like lumen and no cartilage rings in the middle trachea (Figure 3D). Although the rigidity of the malacic trachea in the distal trachea increased with growth (Figures 3E,F), the trachea was still obstructed. The stent was replaced again. Considering that the middle lumen was still collapsed due to the absence of cartilage rings, surgical resection was suggested. The patient's guardians rejected treatment, fearing the risks and the uncertain consequences of the operation. Presently, the child is 3 years and 10 months old, with normal development. He is mostly asymptomatic, experiencing wheezing with mild tachypnea after a respiratory infection. The patient is still being followed up due to a risk of tracheal stent migration and blockage of sputum until stent removal.

## Literature Review

Seven studies that included a total of 12 patients were reviewed. Among them, there were two female and 10 male patients. Three cases were premature. Onset age ranged from birth to 6 months. Six cases had short localized stenosis, one case was of distal tracheal stenosis and tracheal collapse, and five cases were of segmental tracheal collapse. Besides that, trifurcate carina was found in half of the cases (1, 4–6).

Six cases of localized stenosis included one cervical tracheal stenosis, one middle tracheal stenosis, three distal tracheal stenoses proximal to the carina, and one in the juxtacarina location (5, 7, 8). The length of stenosis ranged from 1 to 2 cm. Bronchoscopy revealed soft stenosis of the trachea; some cases mimicked complete tracheal rings. All the six patients underwent surgery, and their operation age ranged from 4 weeks to 16 months. Prognosis was good in all the patients after the operation, even though some of them required dilatation due to restenosis. Three cases reported pathological results, showing irregular plates or almost absence of cartilage. The length of the lesion was unclear in one case with distal tracheal stenosis and tracheal collapse. Resection and slide tracheoplasty were performed due to recurrent episodes of life-threatening events. Histopathology showed reduced mural cartilage and irregular, disconnected cartilaginous rings. CPAP via a tracheostomy tube was administered for more than 12 months after surgery due to persistent bronchomalacia in the patient (7).

There were five patients with segmental deficient cartilage rings that are characteristic of tracheal collapse, including two patients with full-length trachea involved and three patients with middle and distal trachea involved. Long-gap EA was combined in one case, while EA and TEF were present in other two cases. Among the two patients with full-length trachea involved, one case was diagnosed with VACTERL syndrome (EA, TEF, VSD, solitary kidney), for which he underwent Palmaz stent implantation. Repeated balloon dilatations were required due to restenosis; cycled antibiotics and respiratory physiotherapy were prescribed due to severe bilateral bronchiectasis (1). The other patient, who was diagnosed with isolated deficient cartilage rings, survived without treatment, manifesting quiet stridor with normal development at 2 years of age (6). In three patients with segmental tracheal collapse, the middle and the distal trachea were involved; those patients were misdiagnosed with tracheomalacia in infancy. As symptoms were not relieved after conservative management or other interventional treatment, deficient tracheal rings were considered; consequently, tracheal resection and anastomosis were performed. The age at operation ranged from 4 to 9 years old. One case reported by Torre and Seymour (1, 4) underwent tracheostomy in infancy after EA/TEF surgery; the tracheostomy tube could be removed until resection of deficient rings and anastomosis was performed at the age of 9 years old. One case received EA repair, aortopexy, and fundoplication with gastrostomy, respectively, during separate surgeries. His recurrent respiratory symptoms did not improve, and optical coherence tomography (OCT) confirmed the absence of tracheal rings. Resection and anastomosis surgery were performed at the age of 4 (1). Santoli (3) reported on a surgery during which up to 50% of the trachea with deficient cartilage rings was removed to control the symptoms of dyspnea and cyanosis, which were initially diagnosed as tracheomalacia. Histopathology of the resection lumen showed a cartilaginous islet and collapsed tracheal lumen. The clinical symptoms were relieved in three children after the operation, and their prognosis was good.

## Discussion

A regular tracheal ring consists of a “C” cartilage ring and a posterior membrane; the proportion between the cartilage and the membranous trachea is about 4.5:1. The rigidity of the cartilage in the tracheal wall prevents complete airway collapse during expiration (9). Congenital deficient cartilage rings are a rare anomaly that could cause airway obstruction. So far, only 12 cases with congenital deficient cartilage rings have been reported. Clinical symptoms may include recurrent pneumonia, stridor, acute life-threatening events with repeating apnea and cyanotic dying spells, and clinical presentations that lack specificity. Consequently, some patients are misdiagnosed with recurrent pneumonia, tracheomalacia, or other diseases (1, 3, 4, 7). The age of onset and the severity are related to the degree, length, and location of deficient cartilage rings. The clinical symptoms seem more severe when up to 50% of the trachea is completely collapsed or in stenosis located on the juxtacarinal trachea.

Through analysis of existing cases, it has been established that congenital deficient tracheal rings are an embryogenesis anomaly that may occur alone or with esophageal atresia, with a higher prevalence in males (15/18). Tracheal cartilage deficiency may be located in the cervical, middle, and distal trachea with different lengths. Among them, the distal trachea seems to be the most common location (1, 3–8). Histological examinations of the resected trachea show a cartilaginous islet or asymmetric, irregularly disconnected cartilaginous rings. There are significant differences between isolated short-segmental absence and segmental deficient cartilage rings with or without EA and TEF. An analysis of five cases reported in this study, as well as of 11 cases with short-segmental deficient cartilaginous rings previously reported in other studies, revealed that all cases were isolated without EA/TEF or vascular ring malformation. In our study cases, bronchoscopy revealed soft concentric stenosis with “gastric cardia” -like changes, which were also confirmed during surgery and should be distinguished from rigid stenosis caused by a complete tracheal ring.

Surgical resection and tracheoplasty have shown to be effective in most patients, yet the clinical characteristics of long-segmental deficient cartilage rings may be similar to congenital tracheomalacia. The deficient cartilage rings are usually located at the middle to the distal trachea or involve the full length and are partly accompanied by EA and TEF. Bronchoscopy of long-segmental deficient cartilage rings shows anterior tracheal collapse with broadened posterior membranous trachea, which looks like “esophageal” and may be easily misdiagnosed as severe tracheomalacia. As the rigidity of the involved trachea does not tend to increase with growth, the patients do not seem to respond to conservative treatment (3).

Chest CT airway reconstruction can indicate stenosis location, degree, and length. The diameter of the lesion may vary from different positive pressures. In addition, dynamic characteristics could be observed under bronchoscopy. Pathological examination is considered as the gold standard for diagnosis; nevertheless, for these patients, surgical specimens are not available before surgery. OCT can provide detailed information on the structural components of the tracheal wall and can detect the absence of cartilage; thus, it is a useful tool for preoperative evaluation (1).

The pathogenesis of deficient cartilage rings is not fully understood. The normal development of the respiratory system is regulated by multiple genes and signaling pathways. Kim et al. found that an Isl1–Nkx2.1 axis regulates a midline epithelial progenitor cell population that, in turn, orchestrates trachea-esophageal separation in *Xenopus* and mouse genetic models (10). Moreover, Que et al. found that impaired bone morphogenetic protein signaling induces EA and TEF with extensive defects in a tracheal cartilage ring formation (11). Sala et al. concluded that disturbed balances of Fgf10 and Shh might explain a subset of human tracheomalacia without TEF or EA (12). Moreover, Arora et al. concluded that the deduction of Tbx4 and Tbx5 causes defects in both the formation of tracheal cartilage rings and the development of tracheal smooth muscle (13). It is possible that the isolated absence of short-segmental deficient tracheal rings and long-segmental deficient tracheal rings associated with or without EA/TEF are different types of cartilage ring hypoplasia caused by different abnormal embryonic development.

According to existing literature, most of the children required treatment; only one case with mild symptoms did not undergo interventional treatment (6). Four patients in our study underwent surgery, and three of them recovered well; one died of severe infection and anastomotic dehiscence. One patient died after rejecting surgery. The surgical choice should be individualized, length, location, and other associated deformities should be considered. When the deficient cartilage rings are localized far away from the glottis and the carina, resection and direct end-to-end anastomosis are easy to perform. Although removal of up to 50% of the tracheal length has shown to be a successful approach in small children (3); when the length of the involved trachea exceeds 50%, the anastomosis seems to be more difficult, resulting in high morbidity of anastomotic dehiscence and restenosis. Reconstruction of the carina is necessary for juxtacarinal locations and bronchial opening involved. Complete excision is not feasible for symptomatic children with full-length involvement. While interventional management needs to be further discussed, external stabilization with graft may be a feasible approach. Intraluminal airway stents are a satisfactory therapeutic option in the management of severe tracheobronchial obstruction. They can be life-saving in extremely sick children whose other treatments failed or are not indicated (14). However, it should be noted that stent placement should not be used as the final treatment for deficient cartilage rings but as an emergency and palliative treatment for children with life-threatening conditions without surgical indications.

The diagnosis and the treatment of deficient cartilage rings require careful perioperative management. Bronchoscopy may be used for preoperative evaluation, postoperative follow-up, obtaining distal airway pathogenic specimens, and managing anastomotic restenosis. Surgical choice, suture skills, infection control, and airway management are essential for a successful treatment.

## Limitations

The diagnosis was based on endoscopic and/or surgical manifestations, but no histopathological examination was performed. Two patients did not undergo surgery, and one consequently died. Fearing the risk and the uncertain consequences of surgery, the guardians of another patient did not want to change treatment because of the stable breathing achieved with stent placement.

## Conclusion

Congenital deficiency of the tracheal rings is a rare malformation, accompanied by persistent or recurrent stridor, wheezing, paroxysmal dyspnea, and cyanosis that manifests as extreme malacia or stenosis. Short-segmental absent cartilage ring is usually isolated and manifests as soft dynamic stenosis, while long-segmental deficient cartilage rings manifest as extreme malacia and may be associated with EA and TEF. Bronchoscopy helps make a diagnosis when dynamic concentric stenosis or complete tracheal collapse without cartilage rings is observed. For symptomatic patients with deficient cartilage rings, resection and tracheoplasty are suggested as the rigidity of the affected trachea does not increase with growth. The interventional methods should be individualized.

## Data Availability Statement

All datasets presented in this study are included in the article/Supplementary Material.

## Ethics Statement

Written informed consent was obtained from the legal guardian for the publication of any potentially identifiable images or data included in this article.

## Informed Consent

All patients have consented to the submission of the case.

## Author Contributions

JM contributed to study design, data collection and analysis, statistical analysis, and manuscript drafting. CM and ZF contributed to study design, data collection and analysis, and manuscript revision. XH, SL, and NL contributed to data collection. WZ contributed to study design and critical revision of the manuscript. All authors contributed to the article and approved the submitted version.

## Conflict of Interest

The authors declare that the research was conducted in the absence of any commercial or financial relationships that could be construed as a potential conflict of interest.
